# Associations Between Alcohol Use, Antiretroviral Therapy Use, and Viral Load Suppression Among People Living with HIV in Rural Central Uganda

**DOI:** 10.1007/s10461-024-04299-x

**Published:** 2024-05-22

**Authors:** Adriane Wynn, Katelyn M. Sileo, Katherine Schmarje Crockett, Rose Naigino, Michael Ediau, Nicolas A. Menzies, Seth C. Kalichman, Rhoda K. Wanyenze, Natasha K. Martin, Susan M. Kiene

**Affiliations:** 1 Division of Infectious Diseases and Global Public Health, University of California, San Diego, La Jolla, CA USA; 2 Department of Public Health, The University of Texas at San Antonio, San Antonio, TX USA; 3 Division of Epidemiology and Biostatistics, School of Public Health, San Diego State University, 5500 Campanile Drive (MC-4162), San Diego, CA 92182 USA; 4 The Herbert Wertheim School of Public Health and Human Longevity Science, University of California San Diego, La Jolla, CA USA; 5 Harvard T.H Chan School of Public Health, Boston, MA USA; 6 Department of Psychological Sciences, University of Connecticut, Storrs, CT USA; 7 Department of Disease Control and Environmental Health, Makerere University School of Public Health, Kampala, Uganda; 8 Institute for Collaboration on Health, Intervention, and Policy, University of Connecticut, Storrs, CT USA

**Keywords:** Alcohol use, HIV, ART, Viral suppression, Uganda, Cross-sectional study

## Abstract

Alcohol use among people living with HIV (PWH) is common and may negatively affect engagement in HIV care. We evaluated the relationships between alcohol use, ART use, and viral suppression among PWH in Uganda. PATH/Ekkubo was a trial evaluating a linkage to HIV care intervention in four Ugandan districts, Nov 2015-Sept 2021. Our analytical sample included: (1) baseline data from individuals not enrolled in the intervention trial (previously diagnosed HIV+); and 12-month follow-up data from the control group (newly diagnosed or previously diagnosed, but not in care). Level of alcohol use was categorized using the Alcohol Use Disorders Identification Test-Consumption (AUDIT-C): none (AUDIT-C = 0), low (women = 1–2, men = 1–3), medium (women = 3–5, men = 4–5), high/very high (6–12). Multivariable logistic regression models evaluated associations between alcohol use, ART use and viral suppression (a viral load of < 20); we also stratified by gender. Among 931 PWH, medium (OR: 0.43 [95% CI 0.25–0.72]) and high/very high (OR: 0.22 [95% CI 0.11–0.42]) levels of alcohol use were associated with lower odds of being on ART. In a sub-sample of 664, medium use (OR: 0.63 [95% CI 0.41–0.97]) was associated with lower odds of viral suppression. However, this association was not statistically significant when restricting to those on ART, suggesting the relationship between alcohol use and viral suppression is explained by ART use. Among men, high/very high, and among women, medium alcohol use levels were associated with lower odds of being on ART and being virally suppressed. Interventions for PWH who use higher levels of alcohol may be needed to optimize the benefits of Uganda’s Universal Test and Treat strategy.

## Introduction

In 2015, the World Health Organization (WHO) endorsed the universal test-and-treat (UTT) policy that recommends antiretroviral therapy (ART) initiation for all people living with HIV (PWH) at diagnosis, regardless of CD4 count [1].In Uganda, a country that faces a generalized HIV epidemic with a national prevalence of 5.2% [2], the UTT strategy was adopted in December 2016 and fully implemented by July 2017 [3]. Since the removal of this important system-level barrier to ART, Uganda achieved UNAIDS 90–90-90 targets, which aimed to have 90% of all PWH know their HIV status, 90% of people diagnosed with HIV receive sustained ART, and 90% of people receiving ART be virally suppressed (1000 copies/mL) [4]. However, gaps in ART coverage and viral suppression persist in certain communities and sub-populations [5], such as younger men, and underlying factors driving the epidemic need to be addressed to achieve UNAID 95–95-95 Fast Track Targets [6, 7].

Alcohol use is an established barrier to services along the HIV care cascade [8, 9]. Across varying settings, alcohol use has been associated with reduced receipt of and adherence to ART [10–12], and viral suppression [13, 14]. A large body of research exists on the relationship between alcohol use and ART adherence, which suggests that alcohol may be detrimental through several mechanisms, including alcohol myopia [15], cognitive impairment [16], and toxicity beliefs [17]. However, there is less research on the relationship between alcohol use and receipt of ART. A 2015 systematic review found seven studies on alcohol use and ART initiation and five were in the United States. Thus, more research is needed on the relationship between alcohol use and receipt of ART in Sub-Saharan Africa.

Among the Fast Track Targets, implementation of UTT likely has the most direct impact on receipt of ART. According to the Andersen healthcare utilization model [7], the UTT policy would fall into a system-level enabling characteristic that directly impacts healthcare access and subsequent use. Other factors that may influence healthcare utilization, such as ART use, are predisposing characteristics, which include individual demographics (e.g. gender) and social characteristics (e.g. education) that determine biological imperatives for health services use as well as attitudes, values, and knowledge; enabling resources (e.g. wealth) that facilitate or hinder health services use; and need (e.g. HIV severity), which relates to individual demand for health services based on perceived or evaluated health state. Previous research has found that alcohol use may directly reduce perceived need for ART as alcohol could be used as a form of “self-medication.” [18] Beliefs about alcohol and ART interactive toxicity may be a predisposing characteristic [17] that delays HIV care seeking [19]. Further, predisposing and enabling characteristics (e.g. gender and wealth) may confound the relationship between alcohol use and access to ART. As the implementation of UTT has changed the characteristics of the population eligible for ART [20], more research is needed on the relationship between alcohol use and ART use post-UTT.

Uganda has one of the highest levels of alcohol use per capita in the world (four times higher than the global average), and over 20% of the population reported heavy episodic drinking (~ 6 standard drinks, at least once in the past month) in 2018 [21]. Further, alcohol use was found to be more common among PWH compared to those without HIV [22]. Most research examining the relationship between alcohol use, ART receipt, and viral suppression has used dichotomous measures of alcohol use. For example, one study assessed the relationship between alcohol use and ART in the UTT era in the Rakai region and found that ART use was more common among those with no reported alcohol use (83.4%) compared to those who reported any alcohol use in the past year past year (75.8%) (p < 0.0001) [5]. However, a more nuanced measure of alcohol use may help identify sub-groups of people who use alcohol that are at higher risk of not achieving HIV care cascade goals. For example, a study among US veterans found that medium and high (but not low) levels of alcohol use were associated with reduced likelihood of being on ART [13]; and a pre-UTT study in Kenya and Uganda found that a very high level of alcohol use (but not low, medium, or high) was associated with reduced likelihood of viral suppression [8].

The current study sought to build on past research by evaluating the relationships between ART use, viral suppression, and self-reported alcohol consumption patterns, using a validated measure of alcohol use, among PWH in rural, Central Uganda after implementation of UTT. As men and women in Uganda may have differences in terms of alcohol consumption and engagement in HIV care, we also evaluated those relationships stratified by gender. Identifying barriers to HIV care and ART is important to ensure the overall success of the UTT policy and that the benefits are equitably distributed.

## Methods

### Setting and Study Design

This study used cross-sectional data from the PATH/Ekkubo matched-pair, two-arm, cluster randomized trial, which tested an intervention to enhance linkage to HIV care and improve viral suppression among people newly diagnosed with HIV. As previously described, the trial involved community-wide, home-based HIV testing and took place in rural districts of Butambala, Mpigi, Mityana, and Gomba in central Uganda [23]. Villages were randomized to the intervention or standard-of-care arm. Study eligibility criteria were: adults aged 18 to 59 years old or emancipated minors aged 15 to 17 years, accepting HIV testing, speaking Luganda or English, and residing in a household within participating villages.

At baseline, home-based HIV counseling and testing was provided to all eligible and consenting participants. All participants at baseline responded to an interviewer-administered, computer-based questionnaire, regardless of HIV status. Baseline interviews occurred between November 2015 and March 2020 and alcohol use questions were added in November 2017. HIV status was assessed by health workers using the World Health Organization (WHO) algorithm for generalized epidemics [24], which started with a finger stick capillary blood sample for an HIV rapid test assay (Alere/Abbott Laboratories, Chiba, Japan). Participants who were newly diagnosed or previously diagnosed and not linked to care were invited to participate in the intervention trial. Those enrolled in the trial and living in villages randomized to the standard-of-care arm received counseling and paper-based referrals that included a list of health facilities with free HIV care, including locations and HIV clinic hours and a second home visit 2 weeks later with their CD4 test result and reinforcement of the referral to care. Those enrolled in the trial also responded to follow-up questionnaires 6- and 12-months after enrollment. A sample for HIV viral load testing was taken via venous blood draw at trial enrollment and 12-month follow up. Follow-up data were collected between November 2016 and September 2021. Participants who were previously diagnosed with HIV and had been linked to care were ineligible to participate in the trial but were invited to participate in a 12-month viral load monitoring arm. Those consenting to participate in the viral load monitoring arm in addition to already completing a baseline interview, provided venous blood samples for viral load testing at baseline and again 12 months later.

**Fig. 1:**
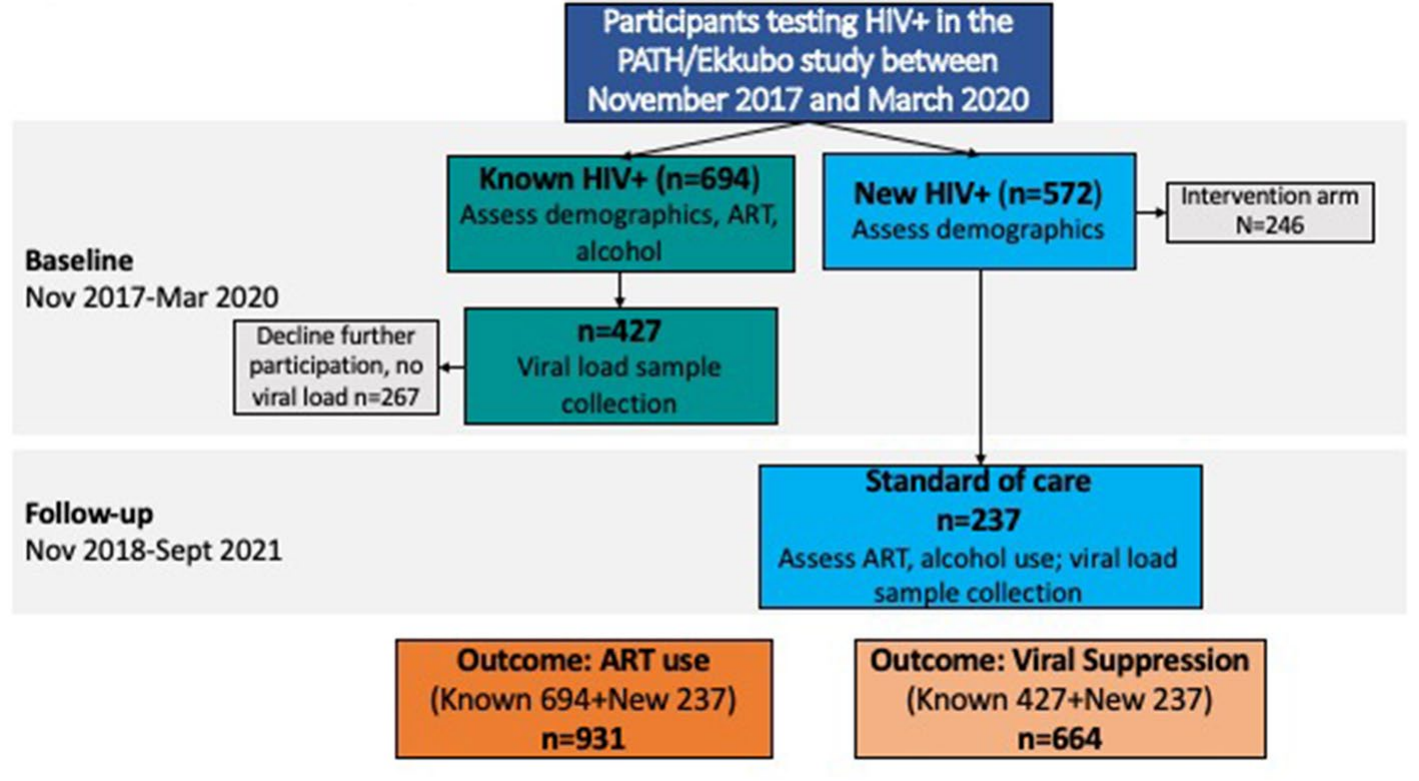
Flow chart for study analytical sample using PATH/Ekkubo data

This study combines data from two groups of participants: 1) Baseline data from participants who were previously diagnosed, and 2) 12-month follow-up data from participants who were newly diagnosed with HIV at baseline and enrolled in the control group (Fig. 1). Thus, our analytical sample was selected to reflect individuals who had been diagnosed with HIV at least 12 months prior, were eligible for ART due to the expansion of UTT, and had access to ART through the standard of care offered in rural, Central Uganda.

### Measures

Alcohol use was the primary regressor of interest. Consumption was assessed using the 3-item Alcohol Use Disorders Identification Test Consumption (AUDIT-C) [25], which estimates the frequency and quantity of alcohol use and can be used to classify people into categories of risk. Scores range from 0–12. Drinking levels were categorized as none (AUDIT-C = 0), low (women = 1–2, men = 1–3), medium (women = 3–5, men = 4–5), high/very high (6–12). These categories have been identified in previous research as meaningful thresholds for differences in risk of adverse HIV care outcomes [8, 13]. We combined high and very high levels of alcohol use because of small cell sizes. Participants were asked to consider beer, wine, malwa, waragi, tonto or any beverage that contains alcohol, but to exclude wine received at church. Within the computerized questionnaire, participants were shown an image of local alcoholic beverages of varying sizes and what constitutes a single drink to help with the accurate reporting of total drinks. Study interviewers used the images to calculate the number of standard drinks based on a participant’s responses about the type, size, and number of specific alcoholic beverages they consume.

The main outcomes were ART use and viral load suppression. ART use was self-reported based on the question, “Are you taking ARVs”? Viral load suppression was defined as having an undetectable viral load of < 20 copies/ml from the sample taken at 12 months for newly diagnosed PLHIV and the sample taken at baseline for previously diagnosed PWH.

Other measures considered in the present analysis that may serve as confounders between alcohol use and ART use and viral suppression [26], included predisposing (e.g. age, gender, marital status, religion) and enabling (e.g. education, household wealth) characteristics and a measure of depressive symptoms, which often co-occur with alcohol use. Socio-demographic information was collected via interviewer-administered questionnaire and included gender, age, educational level, religion, marital/partnership status (not married included those never married, separated and divorced), and socioeconomic status (measured using a wealth index). The wealth index was created based on questions from the Uganda Demographic Health Survey. A factor analysis was conducted using seven household characteristics (e.g. roof and floor materials, and whether the household had electricity, television, or a sofa set) and household scores were distributed among quintiles. Depressive symptoms were assessed using a modified 10-item version of the Center for Epidemiological Studies of Depression Short Form (CES-D-10) scale, an abbreviated version of the original 20-item scale [27]. The scale has been shown to be reliable in a rural Ugandan sample (α = 0.90) [28]. We used a threshold of 14 or higher to identify those at risk for depression, which followed previous research showing a need for a higher cut-off score in populations in Sub-Saharan Africa [29].

### Analysis

All analyses were conducted using Stata Statistical Software: Release 17 (College Station, TX). First, we explored participant characteristics and alcohol use with descriptive statistics. Potential covariates were identified based on our conceptual model and associations found in previous literature [26], and included: age, gender, marital status, religion, education, wealth, depressive symptoms score, and study group (i.e. known HIV positive at baseline or 12 month follow-up among newly diagnosed). Variables were included in the final model based on subject matter significance, relationships among the independent variables, and statistical significance. Statistical significance was determined based on an alpha level of 0.05 for covariates and 0.10 for interactions. As depression has been identified as a comorbid syndrome with alcohol use [30], the interaction between alcohol use and CES-D-10 variable was tested and found not to improve the fit of any of the models. For each regression model, we tested whether the relationship between level of alcohol use and each binary outcome was linear using orthogonal polynomial contrast tests. We also estimated the adjusted predicted prevalence and 95% confidence intervals of being on ART and virally suppressed for each level of alcohol use. Additionally, we assessed the relationship between alcohol use levels and viral suppression among participants on ART as well as stratified all analyses by gender (men/women) and by sub-sample (previously diagnosed at baseline and newly diagnosed with outcomes collected at 12-month follow-up). We also assessed the missingness in the analytical sample used to examine alcohol use and viral load suppression.

We conducted a reviewer-suggested sensitivity analysis that examined whether participants not on ART had ever received HIV care and/or treatment.

## Results

Between November 2017 and September 2021, 931 HIV-positive adults were eligible for the study and completed the alcohol screening questions as part of the baseline assessment, including 694 in the known HIV-positive PWH group and 237 in the newly diagnosed HIV-positive group (including five previously diagnosed, but not linked to care). From the known HIV-positive PWH group, 265 declined participation in the longitudinal viral load monitoring, thus the analytical sample for the viral load outcome was 664. An analysis comparing those who declined participation in the longitudinal viral load monitoring to those who agreed found that those who participated and who declined were similar in terms of age, gender, marital status, education, religion, wealth, depressive symptoms, and any alcohol use in the past year. However, those who declined participation in viral load monitoring were more likely to report being on ART (95% compared to 90%; p value = 0.009).

Characteristics of the analytical sample for the outcome of ART use and the analytical sample for the outcome of viral suppression are described in Table 1. In the ART outcome sample (n = 931), 86% reported that they were on ART at the time of the interview. In the viral suppression outcome sample (n = 664), 62% had an undetectable viral load. In both the ART outcome and viral suppression outcome samples, the known HIV-positive PWH group made up the majority of participants; half of each of the samples were 35 years of age or older, and most were female, married, achieved a primary level of education, were in the lower levels of the wealth index, and were Catholic. Depressive risk (≥ 14 on the CES-D-10 index) was common (17% in the ART outcome sample and 16% in the viral suppression sample). In terms of alcohol use, just over half reported no alcohol use in both analytic samples (54% in ART sample, 56% in viral load sample). In bivariate comparisons, being in the known HIV-positive group, of older age, female, and married were characteristics associated with being on ART and viral suppression. Alcohol use was negatively associated with being on ART, but this relationship was not statistically significant for viral load suppression.

**Table 1:** Characteristics of the analytical samples of PWH in rural, central Uganda between November 2017-March 2020: sample for the antiretroviral treatment (ART) outcome and sample for HIV viral suppression outcome

Characteristics	Sample for ART outcome				Sample for viral suppression outcome			
	Total	No	Yes		Total	No	Yes	
	N = 931	N = 133	N = 798		N = 664	N = 254	N = 410	
	N (%)			p value	N (%)			p value
Group				< 0.001				< 0.001
Known HIV+ (baseline data)	694 (75%)	55 (41%)	639 (80%)		429 (65%)	130 (51%)	299 (73%)	
New HIV+ (12-month follow-up data)	237 (25%)	78 (59%)	159 (20%)		235 (35%)	124 (49%)	111 (27%)	
Age (years)				< 0.001				< 0.001
15/24	155 (17%)	45 (34%)	110 (14%)		125 (19%)	67 (26%)	58 (14%)	
25/34	303 (33%)	39 (29%)	264 (33%)		211 (32%)	93 (37%)	118 (29%)	
35+	473 (51%)	49 (37%)	424 (53%)		328 (49%)	94 (37%)	234 (57%)	
Gender				0.002				< 0.001
Male	243 (26%)	49 (37%)	194 (24%)		194 (29%)	94 (37%)	100 (24%)	
Female	688 (74%)	84 (63%)	604 (76%)		470 (71%)	160 (63%)	310 (76%)	
Marital				< 0.001				0.014
Not married	394 (42%)	57 (43%)	337 (42%)		286 (43%)	113 (44%)	173 (42%)	
Married	537 (58%)	76 (57%)	461 (58%)		378 (57%)	141 (56%)	237 (58%)	
Education				0.51				0.45
None	95 (10%)	14 (11%)	81 (10%)		71 (11%)	21 (8%)	50 (12%)	
Primary	642 (69%)	89 (67%)	553 (69%)		447 (67%)	176 (69%)	271 (66%)	
Secondary and above	194 (21%)	30 (23%)	164 (21%)		146 (22%)	57 (22%)	89 (22%)	
Wealth Index Score				0.18				0.36
1	393 (42%)	43 (32%)	350 (44%)		258 (39%)	95 (37%)	163 (40%)	
2	254 (27%)	42 (32%)	212 (27%)		186 (28%)	67 (26%)	119 (29%)	
3	78 (8%)	14 (11%)	64 (8%)		62 (9%)	28 (11%)	34 (8%)	
4	140 (15%)	23 (17%)	117 (15%)		102 (15%)	37 (15%)	65 (16%)	
5	66 (7%)	11 (8%)	55 (7%)		56 (8%)	27 (11%)	29 (7%)	
Religion				0.52				0.73
Protestant	214 (23%)	32 (24%)	182 (23%)		167 (25%)	59 (23%)	108 (26%)	
Catholic	495 (53%)	67 (50%)	428 (54%)		333 (50%)	130 (51%)	203 (50%)	
Moslem	127 (14%)	16 (12%)	111 (14%)		93 (14%)	39 (15%)	54 (13%)	
Other	95 (10%)	18 (14%)	77 (10%)		71 (11%)	26 (10%)	45 (11%)	
Depressive symptoms				0.46				0.35
CES-D-10 < 14	770 (83%)	113 (85%)	657 (82%)		556 (84%)	217 (85%)	339 (83%)	
CES-D-10 ≥ 14	161 (17%)	20 (15%)	141 (18%)		108 (16%)	37 (15%)	71 (17%)	
Alcohol use levels				< 0.001				0.559
None	507 (54%)	61 (46%)	446 (56%)		373 (56%)	134 (53%)	239 (58%)	
Low	226 (24%)	26 (20%)	200 (25%)		144 (22%)	58 (23%)	86 (21%)	
Medium	119 (13%)	23 (17%)	96 (12%)		88 (13%)	37 (15%)	51 (12%)	
High/very high	79 (8%)	23 (17%)	56 (7%)		59 (9%)	25 (10%)	34 (8%)	

Alcohol use levels: none: AUDIT-C = 0, low: AUDIT-C 1–3 men/1–2 women, medium: AUDIT-C 4–5 men/3–5 women, high: AUDIT-C 6–7, very high: AUDIT-C 8–12

p values were derived from Chi-squared tests

**Fig. 2:**
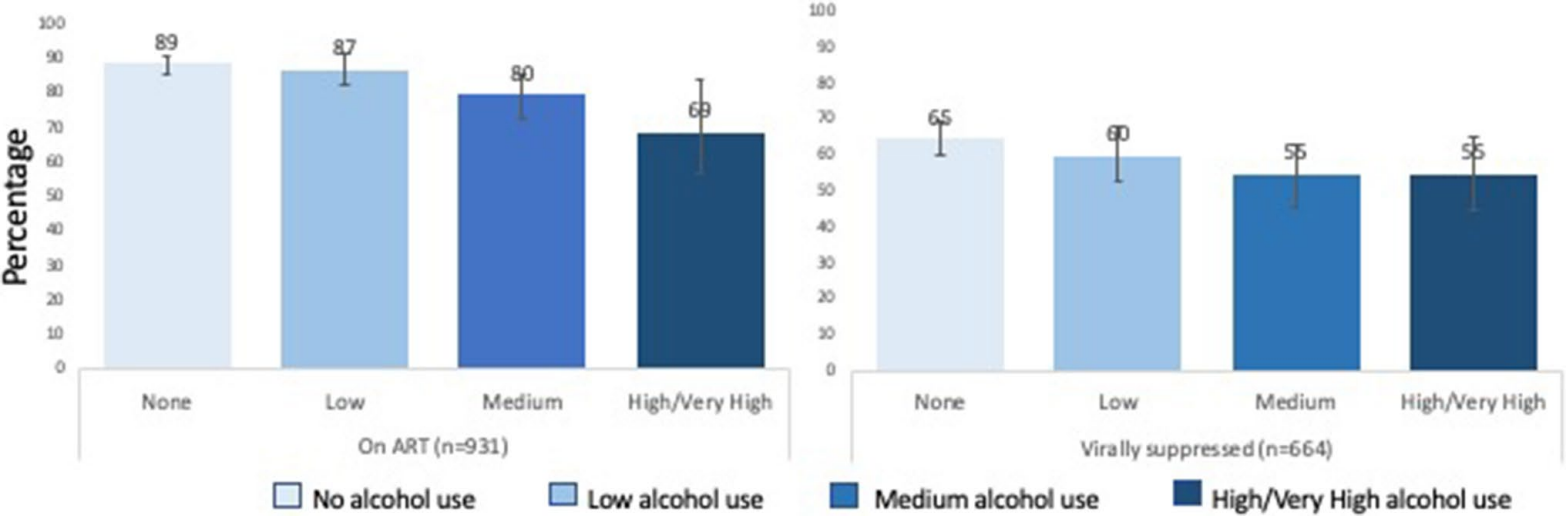
Predicted prevalence of being on ART and virally suppressed by alcohol use level. “On ART” model was adjusted for age, gender, marital status, education, and sample/time point. “Virally Suppressed” model was adjusted for age, gender, religion, and sample/time point

In adjusted models, compared to no alcohol use in the past year, medium (aOR = 0.43; 95% CI 0.25–0.72; p < 0.001) and high/very high (aOR = 0.22; 95% CI 0.11–0.42; p < 0.001) levels of alcohol use decreased the odds of being on ART and the linear trend was statistically significant (p < 0.001) (Table 2). However, only medium alcohol use (aOR = 0.63; 95% CI 0.41–0.97; p = -0.035) was associated with a decreased likelihood for viral load suppression, compared to no alcohol use, and a linear trend was not found (p = 0.175). Further, when we restricted the sample to participants on ART, we found no statistically significant relationship between alcohol use level and viral suppression (Table 3). The adjusted predicted prevalence of ART use and viral suppression are found in Fig. 2. The adjusted prevalence estimates for being on ART and achieving viral load suppression were < 90% for all levels of alcohol use. The prevalence of being on ART reduced linearly as levels of alcohol use increased. In models stratified by gender (Table 4) we found that, among men, high/very high levels of alcohol use were associated with lower odds of being on ART (aOR = 0.07; 95% CI 0.02–0.23; p < 0.001) and of being virally suppressed (aOR = 0.20; 95% CI 0.09–0.48; p < 0.001). Among women, medium levels of alcohol use were associated with lower odds of being on ART (aOR = 0.38; 95% CI 0.19–0.75; p = 0.005) and virally suppressed (aOR = 0.53; 95% CI 0.30–0.98; p = 0.038). Table 5 provides the results of models stratified by sub-sample. High/Very high levels of alcohol use were associated with reduced odds of being on ART among those previously diagnosed (aOR = 0.16; 95% CI 0.08–0.36; p =  < 0.001) and those newly diagnosed (aOR = 0.25; 95% CI 0.07–0.93; p = 0.039) at enrollment (with alcohol use and outcomes collected at 12-month follow-up). Medium levels (aOR = 0.32; 95% CI 0.16–0.62; p < 0.001) were associated with being on ART among the participants who had been previously diagnosed, but not among those newly diagnosed). In terms of viral suppression, medium levels of alcohol use were associated with lower odds of suppression among those newly diagnosed; however, the relationship wasn’t statistically significant among those previously diagnosed.

**Table 2:** Associations between level of alcohol use and two measures of the HIV care cascade from a population-based sample in rural, central Uganda

	OR [95% CI]	p value	Test for linear trend
On ART (n = 931)			
None	*Referent*		< 0.001
Low	0.87 [0.49–1.53]	0.626	
Medium	0.43 [0.25–0.72]	0.001	
High/very high	0.22 [0.11–0.42]	< 0.001	
Viral suppression (n = 664)			
None	*Referent*		0.175
Low	0.80 [0.54–1.19]	0.269	
Medium	0.63 [0.41–0.97]	0.035	
High/very high	0.64 [0.38–1.09]	0.098	

Odds ratios, confidence intervals and p-values derived from multivariable logistic regression models with robust standard errors and adjustment for village clustering. “On ART” model was adjusted for age, gender, marital status, education, and sample/time point. “Virally Suppressed” model was adjusted for age, gender, religion, and sample/time point. ART use was assessed at the time of interview (“Are you currently on ART”). Viral suppression = undetectable viral load of < 20 copies/ml

*OR* odds ratio, *ART* antiretroviral treatment

**Table 3:** Associations between levels of alcohol use and viral suppression among participants on ART (n = 577)

	OR [95% CI]	p value	Test for linear trend
None	Referent		< 0.652
Low	0.73 [0.46–1.17]	0.192	
Medium	0.88 [0.49–1.59]	0.674	
High/very high	1.16 [0.49–2.76]	0.737	

Model was adjusted for age, gender, religion, and sample

**Table 4:** Associations between level of alcohol use and the HIV care cascade stratified by women and men

	Men		Women	
	OR [95% CI]	p value	OR [95% CI]	p value
On ART	n = 243		n = 688	
None	*Referent*		*Referent*	
Low	0.84 [0.33–2.22]	0.739	0.73 [0.37–1.44]	0.362
Medium	0.41 [0.15–1.16]	0.093	0.38 [0.19–0.75]	0.005
High/very high	0.07 [0.02–0.23]	< 0.001	0.45 [0.16–1.20]	0.126
Viral suppression	n = 194		n = 470	
None	*Referent*		*Referent*	
Low	0.67 [0.40–1.12]	0.124	0.83 [0.81–1.54]	0.556
Medium	0.89 [0.39–2.01]	0.781	0.53 [0.30–0.98]	0.038
High/very high	0.20 [0.09–0.48]	< 0.001	2.40 [0.85–6.76]	0.097

Models were adjusted for age, marital status, education, religion, and sample. On ART was measured through self-reported ART use at the time of interview

**Table 5:** Associations between level of alcohol use and the HIV care cascade stratified by study sub-sample

	Known HIV positive (data collected: baseline)		Newly diagnosed/not in care (data collected: 12-month follow-up)	
	OR [95% CI]	p value	OR [95% CI]	p value
On ART	n = 694		n = 232	
None	*Referent*		*Referent*	
Low	0.75 [0.25–2.23]	0.605	1.05 [0.66–1.68]	0.833
Medium	0.32 [0.16–0.62]	0.001	0.50 [0.18–1.40]	0.185
High/very high	0.16 [0.08–0.36]	< 0.001	0.25 [0.07–0.93]	0.039
Viral suppression	n = 429		n = 235	
None	*Referent*		*Referent*	
Low	0.65 [0.38–1.14]	0.133	0.96 [0.51–1.80]	0.556
Medium	0.71 [0.40–1.26]	0.241	0.44 [0.20–0.98]	0.044
High/very high	0.55 [0.29–1.03]	0.061	0.64 [0.26–1.59]	0.335

Models were adjusted for age, marital status, education, religion, and sample. On ART was measured through self-reported ART use at the time of interview

In a sensitivity analysis, we found the majority (62%) of participants not on ART reported they had never received HIV care or treatment; however, this relationship differed between the sub-samples. Among the 78 participants who were newly diagnosed at enrollment and not on ART at 12 months follow-up, 67 (86%) said they had never received medical care or treatment for HIV since the last interview (11 had received HIV care). However, among the 55 previously diagnosed who were not on ART at enrollment, 17 (31%) said they had never received medical care or treatment for HIV, and 38 (69%) had ever been in care.

## Discussion

Among the study sample of PWH in rural, Central Uganda after implementation of UTT, we found that participants who reported medium and high/very levels of alcohol use were significantly less likely to report currently taking ART, compared to those who did not use alcohol. We also found that medium levels of alcohol use decreased the likelihood of being virally suppressed, compared to no alcohol use. Among participants on ART, the association between alcohol use and viral suppression was no longer significant, suggesting that the relationship between alcohol use and non-suppression in the full sample may be explained by lower ART use among those who drink. While previous studies have found that alcohol use is associated with decreased ART adherence [10], our study’s finding that alcohol use was not associated with viral suppression among people on ART may reflect improvements in ART regimens that are less burdensome (e.g. one tablet daily) and have lower requirements for optimal adherence needed for viral suppression, thereby, potentially reducing the negative impact of alcohol use on adherence and subsequently viral suppression [31].

Findings support previous studies in Uganda that found that alcohol use was associated with lower engagement in the HIV care cascade. One study among people seeking HIV care in Mbarara found that abstaining from alcohol was associated with increased likelihood of ART receipt [32]. Two studies taking place in Uganda and Kenya found that any drinking was associated with reduced likelihood of ART initiation [8, 33] and higher levels of alcohol use were associated with lower likelihood of retention in HIV care [33] and viral suppression [8]. Further, the negative association between alcohol use and being on ART that was found in our study was similar to the relative risk found by Puryear et al. 2019 (RR: 0.93; 95% CI 0.89–0.97) in their analysis using data prior to UTT implementation. Finally, a recent paper among participants in the Rakai Community Cohort Study in Uganda post UTT implementation found that people with no past year alcohol use had higher rates of current ART use than those who used alcohol [5]. Those researchers also found that while alcohol use was associated with viral suppression, the relationship was no longer significant after restricting to participants on ART.

We found differences between men and women in terms of the levels of alcohol use that were associated with reduced odds of being on ART or virally suppressed. In Uganda, women and men have different patterns of drinking in general, with men consuming higher quantities of alcohol more frequently [21]. Qualitative work in Uganda suggests it is more socially acceptable for men to drink recreationally and alcohol use may enhance perceptions of masculinity [19]. For women, drinking in public areas or in higher quantities are associated with different risks, such as vulnerability to sexual aggression or violence [34]. Further, gender is a predisposing factor for healthcare utilization with a large body of research finding that men are less likely to utilize healthcare services in general, and men in Uganda are less likely to receive HIV care [35]. Some studies have found that lower ART initiation may also be impacted by issues of masculinity. A study among men on ART in Wakiso District, found that HIV status undermined social capital initially and prevented linkage to care and ART initiation; however, men in that study were able to adjust their new masculine identity and better manage HIV [34]. Thus, issues of masculinity could predispose men to increased alcohol use and reduced ART use.

Prior research in Uganda has also found the relationship between alcohol use and HIV care differed by gender. A recent paper in Rakai found the relationships between alcohol use and the HIV care cascade differed between men and women [5]. A negative relationship between alcohol use consequences (ever experiencing the following during or after drinking: 1. had an unsteady gait; 2. fell over; 3. got angry; 4. got violent; 5. had difficulty speaking; 6. forgot things that happened; 7. had shaking hands; 8. felt ashamed),and viral suppression was only found among men. Further, the authors found that the negative relationship between any alcohol use (vs no alcohol use) and viral suppression was mainly driven by women. Together this research may suggest that women experience adverse HIV care outcomes at lower levels and men at higher levels of alcohol use; however, more research is needed to understand these relationships.

We also found differences between participants who were newly diagnosed at baseline and those who had been previously diagnosed. Alcohol use appeared to be a barrier to being on ART in both study groups. However, the negative relationship between alcohol use and viral load suppression was only significant among those newly diagnosed, signaling that services to address higher levels of alcohol use could be particularly beneficial for individuals with a new HIV diagnosis.

Evidence supporting a negative relationship between alcohol and ART use is growing; however, the mechanisms are less understood. Beliefs about alcohol and ART interactive toxicity are common [17] and could present a barrier to those worried about pressure to reduce alcohol consumption. One study among fisherfolk in Uganda found that men did not initiate ART until they were able to reduce drinking on their own [19]. Previous research also found that healthcare providers can discourage ART among those who drink alcohol [19, 36]. Alternatively, some research suggests that the impact of alcohol use on HIV care could be confounded by predisposing characteristics, including delayed reward discounting [37]. For example, individuals with higher discounting of delayed rewards may have a high preference for the immediate rewards of alcohol use and a lower preference for the benefits of ART uptake, which are realized further into the future [37]. Future research is needed to explore mechanisms underlying the associations between alcohol use and HIV care.

Although systemic barriers to HIV care and ART have been reduced in Uganda, interventions for PWH who use medium to very high levels of alcohol are needed to ensure optimal ART use, viral suppression, and reduced HIV transmission and mortality. A systematic review and meta-analysis identified 21 studies that evaluated behavioral interventions to address alcohol use among PWH and found they were overall successful at reducing alcohol and increasing ART adherence [38]. The majority of studies addressed alcohol use as a component of a multiple HIV behavior change intervention. Another systematic review and meta-analysis of psychosocial interventions conducted in Sub-Saharan Africa found that interventions increased alcohol abstinence at 3–6 months and 12–60 months, but had no impact on AUDIT scores, drinks per drinking day or percentage of drinking days [39]. While some effective interventions exist, the reviews highlighted the need to expand beyond individual-level psychosocial approaches to address structural drivers of alcohol use. Further, if effective and cost-effective interventions to reduce alcohol use among PWH are not available, PWH who use alcohol at higher levels could be prioritized for services to ensure linkage to care and initiation and sustained ART.

Our study has several limitations. First, the alcohol use measure, the AUDIT-C, is based on self-report and drinking levels may be underreported as a result of social desirability and recall biases [40]. For example, bias could be introduced if participants not yet on ART were more likely to under-report their alcohol consumption for fear of being denied treatment in the future or underreporting among those on treatment as they may be told not consume alcohol while on ART. However, we believe this bias is low as our sub-study did not include participants in the parent study’s intervention arm, interviews were conducted outside of health facilities (in participants’ homes), and the interview administrators were not involved in linkage to care or treatment decisions. Future studies would be strengthened with the use of objective biological markers and other techniques to reduce social desirability bias [41]. Second, non-standard drink sizes and alcohol concentrations may cause misclassification of alcohol use levels. However, care was taken to provide images of local alcoholic beverages to guide interviewers in inquiring about drinking and in calculating standard drinks. Third, alcohol use patterns were assessed in reference to the past year, but ART use and viral load suppression were assessed on the day of the interview. Thus, our study was unable to assess the time-varying nature of alcohol use, ART use, and viral load. Further, although our sensitivity analysis found that the majority of participants not on ART had never engaged in HIV care, our variable did not differentiate between ART use and ART adherence. It is possible that participants who were not using ART, but had previously initiated care, were actually on ART and had suspended treatment. Fifth, the analytical sample used to examine the relationship between alcohol use and viral load suppression was missing participants who declined viral load monitoring. While those missing viral load results were similar to the sample with results on many characteristics, they were more likely to be on ART. Therefore, results may not be generalizable to the general population of PWH in rural, Central Uganda. Further, as women tend to drink less than men, among women, the cell sizes for high/very high alcohol use were also small and may explain the lack of relationship at this level. Finally, this analysis used cross-sectional data and therefore is unable to assess causation nor explicitly examine the difference in the impact of alcohol use on ART and viral suppression before and after UTT implementation.

In conclusion, we found that higher levels of alcohol use were associated with a decreasing likelihood of being on ART among a sample of PWH in rural, Central Uganda. We also found that medium levels of alcohol use were associated with reduced odds of viral load suppression. However, this relationship was no longer significant when restricting our sample to those on ART, suggesting that the relationship between alcohol use and non-suppression in the full sample may be explained by lower ART use among those who drink. Despite implementation of UTT and the potential for all to access ART following an HIV diagnosis, participants who drank at higher levels were less likely to be on ART. Thus, more research is needed to identify effective alcohol reduction interventions and PWH who drink alcohol at higher levels should be considered for prioritized linkage and retention services.
